# Both TRIM5α and TRIMCyp have only weak antiviral activity in canine D17 cells

**DOI:** 10.1186/1742-4690-4-68

**Published:** 2007-09-24

**Authors:** Julie Bérubé, Amélie Bouchard, Lionel Berthoux

**Affiliations:** 1 Laboratory of Retrovirology, GRBCM, University of Québec, Trois-Rivières, QC G9A 5H7, Canada

## Abstract

**Background:**

TRIM5α, which is expressed in most primates and the related TRIMCyp, which has been found in one of the New World monkey species, are antiviral proteins of the TRIM5 family that are able to intercept incoming retroviruses early after their entry into cells. The mechanism of action has been partially elucidated for TRIM5α, which seems to promote premature decapsidation of the restricted retroviruses. In addition, through its N-terminal RING domain, TRIM5α may sensitize retroviruses to proteasome-mediated degradation. TRIM5α-mediated restriction requires a physical interaction with the capsid protein of targeted retroviruses. It is unclear whether other cellular proteins are involved in the inhibition mediated by TRIM5α and TRIMCyp. A previous report suggested that the inhibition of HIV-1 by the rhesus macaque orthologue of TRIM5α was inefficient in the D17a canine cell line, suggesting that the cellular environment was important for the restriction mechanism. Here we investigated further the behavior of TRIM5α and TRIMCyp in the D17 cells.

**Results:**

We found that the various TRIM5α orthologues studied (human, rhesus macaque, African green monkey) as well as TRIMCyp had poor antiviral activity in the D17 cells, despite seemingly normal expression levels and subcellular distribution. Restriction of both HIV-1 and the distantly related N-tropic murine leukemia virus (N-MLV) was low in D17 cells. Both TRIM5α_rh _and TRIMCyp promoted early HIV-1 decapsidation in murine cells, but weak levels of restriction in D17 cells correlated with the absence of accelerated decapsidation in these cells and also correlated with normal levels of cDNA synthesis. Fv1, a murine restriction factor structurally unrelated to TRIM5α, was fully functional in D17 cells, showing that the loss of activity was specific to TRIM5α/TRIMCyp.

**Conclusion:**

We show that D17 cells provide a poor environment for the inhibition of retroviral replication by proteins of the TRIM5 family. Because both TRIM5α and TRIMCyp are poorly active in these cells, despite having quite different viral target recognition domains, we conclude that a step either upstream or downstream of target recognition is impaired. We speculate that an unknown factor required for TRIM5α and TRIMCyp activity is missing or inadequately expressed in D17 cells.

## Background

TRIM5α is a primate protein expressed in the cytoplasm of many cell types that is able to inhibit ("restrict") the replication of selected retroviruses [1-3]. Individual *TRIM5α *alleles are able to restrict a few or many retroviruses (although never all of them). The specificity of the restriction, i.e. the viral targets for each particular *TRIM5α *allele, is species-dependent more than it is cell type-dependent. The specific recognition of viral targets is determined by the SPRY/B30.2 region at the C-terminus of TRIM5α [4-9]. On the virus side, capsid (CA) proteins seem to be the only determinant of sensitivity to TRIM5α [10-12], and a physical interaction takes place between TRIM5α and capsid, as evidenced by pull-down assays [13,14]. It is worth noting, however, that the interaction has not yet been documented using purified TRIM5α, and thus it is possible that other cellular factors are relevant to this step. TRIM5α forms trimers and possibly multimers of higher orders of complexity [15,16]. TRIM5α multimerization is linked to its restriction activity [17]. In addition, TRIM5α targets multimers of properly maturated and assembled retroviral CA constituting the capsid core of incoming viral particles [18,19]. Thus, the initial TRIM5α-retrovirus interaction might involve the assembly of a multimer of TRIM5α around the capsid core of incoming retroviruses very early after entry.

Following this initial interaction, replication of the restricted retrovirus can be impaired in several ways. First, TRIM5α_rh _and TRIM5α_hu _seem to promote premature decapsidation of HIV-1 and N-tropic murine leukemia virus (N-MLV), respectively [14,20]. More specifically, TRIM5α causes post-entry disappearance of CA in its particulate form, which is assumed to belong to not-yet-disassembled viruses. Second, replication is inhibited by a mechanism involving the proteasome. This is evidenced by the partial rescue of retroviral replication from TRIM5α restriction in the presence of the proteasome inhibitor MG132 [21,22]. In addition, the ubiquitin ligase activity associated with the RING domain of TRIM5α is important for full restriction activity [3]. It has also been proposed recently that TRIM5α_rh _might promote the degradation of HIV-1 CA through a non-proteasomal, non-lysosomal pathway [23]. Thirdly, TRIM5α interferes with the nuclear transport of retroviral pre-integration complexes [21,22]. TRIM5α from the squirrel monkey seems to restrict the mac251 strain of simian immunodeficiency virus (SIVmac251) mostly, if not only, by inhibiting this nuclear transport step [24].

Interestingly, a recent report pointed to late steps (i.e. assembly and release) of retroviral replication as possibly targeted by TRIM5α, although the molecular basis for late-stage restriction specificity is distinct from that of early stages [25].

In the owl monkey, a New World species, the SPRY/B30.2 domain of TRIM5α is replaced by the full coding sequence of the highly conserved, ubiquitously expressed peptidyl-prolyl isomerase Cyclophilin A (CypA), yielding a protein called TRIMCyp or TRIM5-CypA [26,27]. TRIMCyp inhibits HIV-1, the African green monkey strain of SIV (SIV_AGM_), feline immunodeficiency virus (FIV) and equine infectious anemia virus (EIAV) [28-30]. CypA was isolated fifteen years ago as a cellular protein interacting with HIV-1 CA [31] and TRIMCyp binds CA through its CypA domain [27,28]. CypA-CA interaction and TRIMCyp-mediated restriction are abrogated in the presence of cyclosporine (CsA), a drug that targets the same structural motif in CypA to which CA binds [27,32]. Like TRIM5α_rh_, TRIMCyp causes an early block to HIV-1 replication, preventing the accumulation of retroviral cDNA in the infected cells [16,28,33]. Prior to the present work, however, it was not known whether TRIMCyp promoted HIV-1 premature decapsidation.

Are other cellular factors important for the restriction mediated by TRIM5α? Efficient inhibition of HIV-1 by TRIM5α in several Old World monkey cell lines requires the presence of CypA, as seen by gene knock-down [34,35]. The proposed model [34] is that CypA catalyzes the cis-trans isomerization of HIV-1 CA at proline 90 [36], thus turning it into a target for some simian TRIM5α orthologues. However, the impact of CypA on the restriction of HIV-1 is much less significant when TRIM5α is over-expressed in non-primate cells [34,35,37]. It is not clear whether other cellular proteins are important in the steps leading to the initial viral recognition step. Downstream of this TRIM5α-target interaction, it is expected that cellular proteins take part in the targeting of restricted viruses to proteasome-dependent degradation, although the exact mechanism has not been elucidated yet. Whether cellular proteins other than TRIM5α are also required for CA premature decapsidation and the inhibition of nuclear transport is totally unknown.

The restriction phenotype stemming from TRIM5α and TRIMCyp activity is retained upon expression of these proteins in non-primate cells such as murine and feline cells, suggesting that if cellular factors other than TRIM5α are required, they must be widely conserved among mammals. However, the Poeschla group recently reported that restriction of HIV-1 by the rhesus macaque TRIM5α orthologue was inefficient in D17 cells, a canine osteosarcoma cell line [38]. As a first step toward the isolation of additional factors involved in the restriction by TRIM5α, we decided to characterize further the restriction phenotype in the D17 cells.

## Results

**Figure 1:**
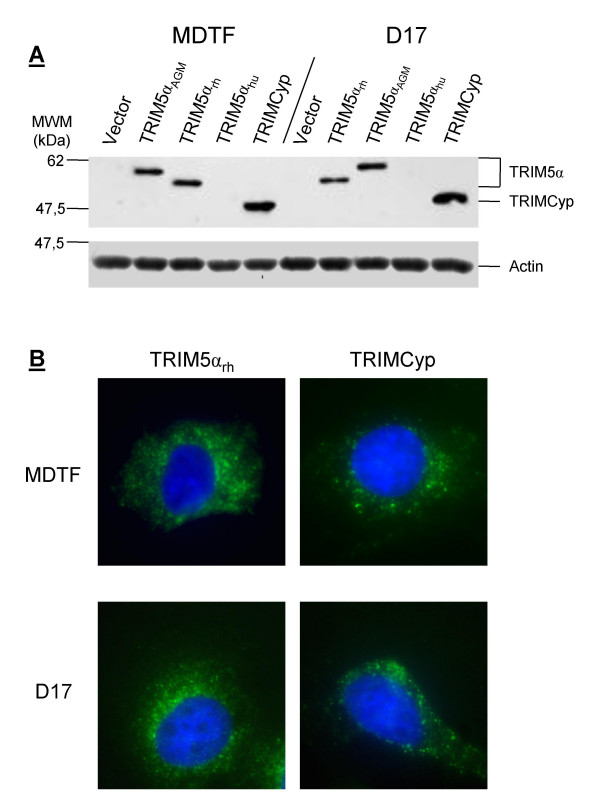
**Expression and subcellular distribution**. **A**, FLAG-tagged TRIM5α (rhesus, human, or African green monkey orthologues) and TRIMCyp were stably expressed in MDTF and in D17 cells, and expression was assessed by western blotting with antibodies directed against FLAG (top) or actin (bottom). The percentage of transduced cells was roughly similar for all cell lines created, as judged by the percentage of puromycin-resistant cells (not shown). The presence of the FLAG tag in TRIM5α_hu _was confirmed by sequencing of the plasmid DNA. **B**, MDTF or D17 cells expressing TRIM5α_rh _or TRIMCyp were fixed and stained using an antibody against FLAG and counterstained with Hoechst33342 to reveal DNA.

**Figure 2:**
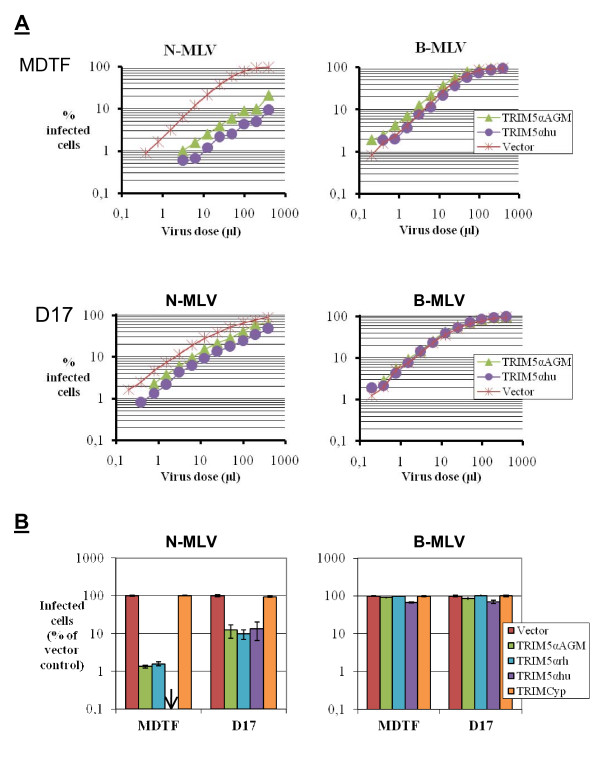
**Restriction of N-MLV**. **A**, MDTF or D17 cells expressing TRIM5α_hu_, TRIM5α_AGM_, or control cells were infected with multiple dilutions of N-MLV and B-MLV vectors expressing GFP. The percentage of infected cells was determined by flow cytometry. **B**, MDTF or D17 cells expressing various orthologues of TRIM5α, or expressing TRIMCyp, were infected with GFP-expressing N-MLV or B-MLV vectors. The virus dose used in each cell type was first adjusted so that 5–10% of control cells would be infected and the same volume of virus preparation was then used to infect the various TRIM5-expressing cell lines from that cell type. The percentage of infected cells was determined 2 days later by flow cytometry, and results are shown as % of the values obtained for the control cells. The experiment was carried out in triplicates, and standard deviations are shown.

We transduced C-terminal FLAG versions of TRIM5α (rhesus macaque, African green monkey, and human) and TRIMCyp (owl monkey) into *mus dunni *tail fibroblasts (MDTF) and D17 cells. Cell lines homogeneously expressing each TRIM5 orthologue were obtained following puromycin treatment. Steady-state levels of TRIM5 expression were similar in MDTF and D17 cells, as judged by western blotting (Fig. 1A). Curiously, we could not detect the human TRIM5α orthologue in either cell line. However, N-tropic murine leukemia virus (N-MLV) was restricted in the MDTF cells expressing TRIM5α_hu _as expected (Fig. 2), and sequencing analysis of pMIP-TRIM5α_hu _confirmed the presence of the FLAG tag. Thus, it appears that TRIM5α_hu_-FLAG has constitutively small steady-state expression levels, an observation previously made by others [39]. We used immunofluorescence (IF) microscopy to analyze the subcellular distribution of TRIM5α_rh _and TRIMCyp in MDTF cells and in D17 cells (Fig. 1B). Both proteins were cytoplasmic and formed bodies in the two cell types. Thus, expression and localization of TRIM5α and TRIMCyp were seemingly normal in the D17 cells.

We then challenged the cell lines generated with N-MLV and B-MLV vectors expressing GFP. Upon infection with multiple virus doses, we found as expected that N-MLV was 10- to 12-fold less infectious in the MDTF cells expressing the human or African green monkey orthologues of TRIM5α, compared with the control cells (Fig. 2A). In the D17 cells, however, the magnitude of restriction by TRIM5α_hu _or TRIM5α_AGM _was only to 2- to 3-fold. As expected, B-tropic MLV replication was not affected by any of the TRIM5α orthologues. In an independent experiment, we infected all the MDTF and D17 cell lines generated with N-MLV_GFP _and B-MLV_GFP _at a single virus dose. TRIM5α_AGM _and TRIM5α_rh _each inhibited N-MLV infection by about 100-fold in the MDTF cells, and TRIM5α_hu _had an even greater inhibitory effect (Fig. 2B). In contrast, restriction in the D17 cells was much smaller (about 10-fold) (Fig. 2B). As expected, N-MLV was not inhibited by TRIMCyp and B-MLV was not inhibited by either TRIM5α  or TRIMCyp.

**Figure 3:**
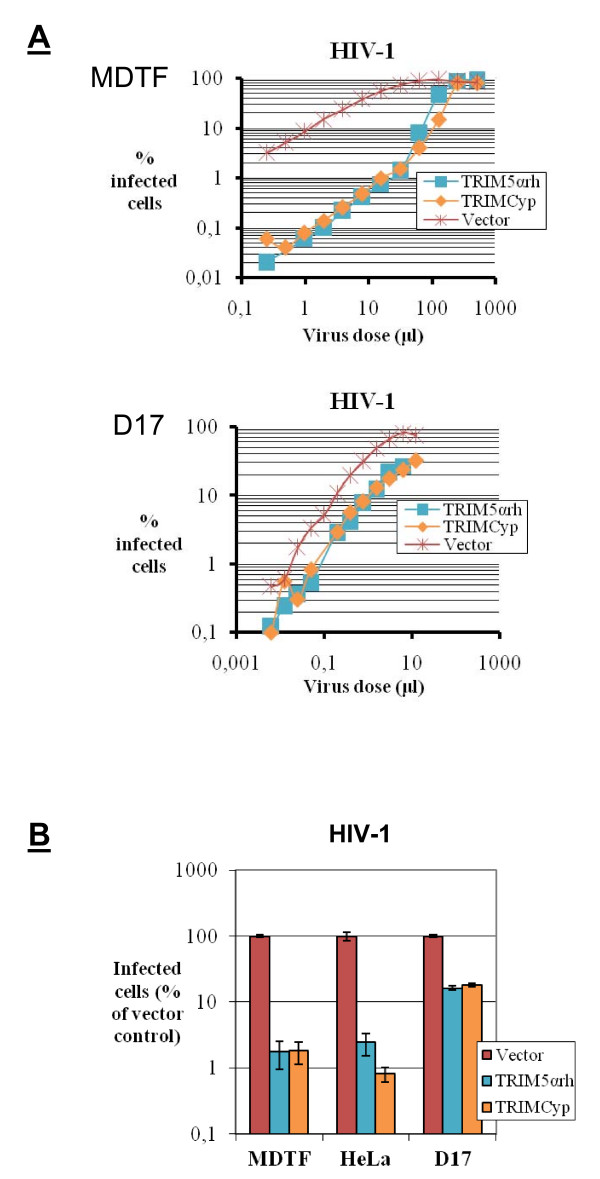
**Restriction of HIV-1**. **A**, MDTF or D17 cells expressing TRIM5α_rh_, TRIMCyp, or control cells were infected with multiple dilutions of HIV-1_GFP_, an HIV-1 vector expressing GFP. The percentage of infected cells was determined by flow cytometry. **B**, MDTF, HeLa or D17 cells expressing TRIM5α_rh _or TRIMCyp, and control cells were infected with HIV-1_GFP_. The virus dose used in each cell type was first adjusted so that 5–10% of control cells would be infected and the same volume of virus preparation was then used to infect the various TRIM5-expressing cell lines from that cell type. The percentage of infected cells was determined 2 days later by flow cytometry, and results are shown as % of the values obtained for the control cells. The experiment was carried out in triplicates, and standard deviations are shown.

We next investigated the levels of restriction of HIV-1 in the various cell lines. Upon challenge at multiple virus doses, we found HIV-1_GFP _to be strongly inhibited (about 100-fold; Fig. 3A) in the MDTF cells expressing either TRIM5α_rh _or TRIMCyp, as expected. In contrast, the level of restriction by these TRIM5 proteins was much smaller in the D17 cells (about 3-fold). In another experiment, we infected MDTF, HeLa, and D17 cells expressing either TRIM5α_rh _or TRIM_Cyp _with HIV-1_GFP _at a fixed virus dose. In these conditions, we found that TRIM5α_rh _and TRIMCyp caused a ≈ 100-fold decrease in infection by HIV-1_GFP _in MDTF or HeLa cells. In D17 cells, however, the decrease in infectivity was of only 5-fold. Thus, restriction of both N-MLV and HIV-1 by either TRIM5α or TRIMCyp was inefficient in the D17 cells.

**Figure 4:**
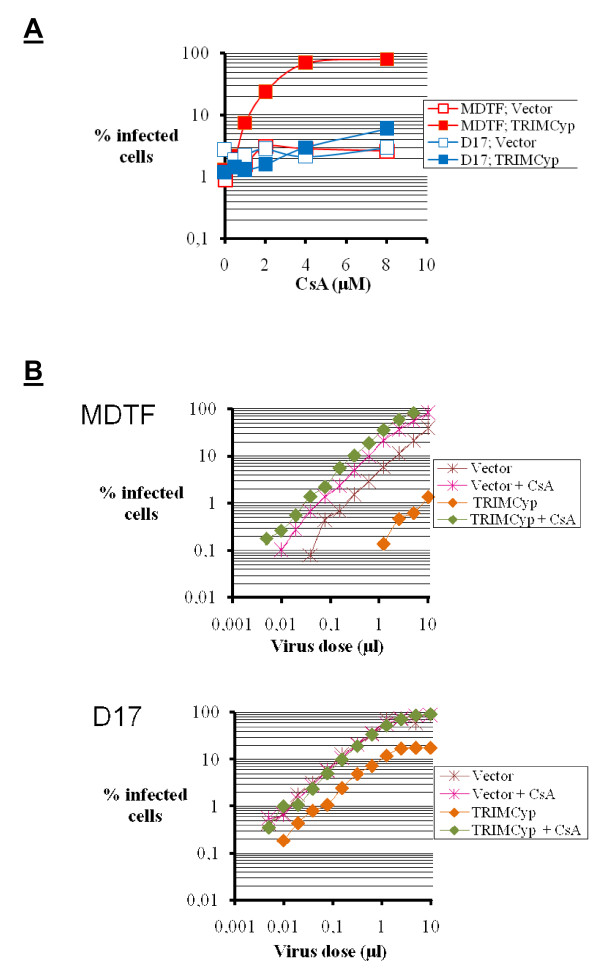
**Enhancement of HIV-1 infection in cells expressing TRIMCyp by cyclosporine**. **A**, MDTF or D17 cells, expressing TRIMCyp or not (control cells), were infected with HIV-1_GFP_. The virus dose was adjusted so that 1% to 3% of cells would be infected in the absence of cyclosporine for each cell line, and the infections were done in the presence of various cyclosporine concentrations. The percentage of infected cells was determined 2 days later by flow cytometry. **B**, as above, except that CsA concentration was constant (6 μM) and cells were infected with multiple doses of HIV-1_GFP_.

Restriction of lentiviruses by TRIMCyp is abrogated in the presence of cyclosporine A (CsA), a competitive inhibitor of cyclophilins. We reasoned that if TRIMCyp inhibited HIV-1 more efficiently in MDTF cells compared to the D17 cells, then the level of enhancement of HIV-1 infection by CsA should also be greater. Thus, we infected MDTF and D17 cells with HIV-1_GFP _at a multiplicity of infection (MOI) of 1 to 3% infected cells and in the presence of increasing CsA concentrations. In MDTF-TRIMCyp cells, CsA enhanced HIV-1 infection by 60-fold (Fig. 4A), consistent with the high level of restriction in these cells. In contrast, CsA-mediated enhancement of HIV-1 replication in D17-TRIMCyp cells was much smaller (5-fold). We performed an additional experiment using an optimal CsA concentration (6 μM) and multiple MOIs (Fig. 4B). CsA completely abrogated TRIMCyp-mediated restriction in both MDTF and D17 cells, but the magnitude of CsA-mediated enhancement of HIV-1 replication was about 20-fold greater in MDTF-TRIMCyp cells compared to D17-TRIMCyp.

**Figure 5:**
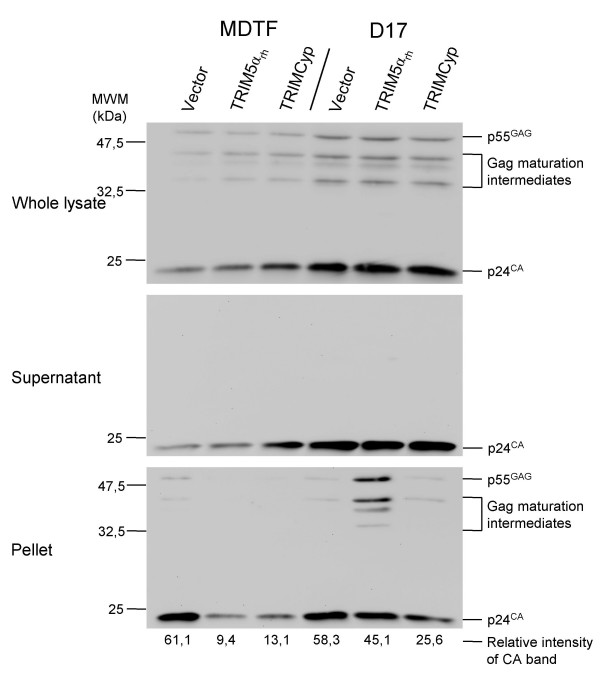
**Fate-of-capsid assay**. MDTF or D17 cells expressing TRIM5α_rh _or TRIMCyp and control cells were infected with HIV-1 VLPs for 4 hours, then cells were allowed to grow for 2 more hours in a virus-free medium. Following the infection, cells were submitted to hypotonic lysis and the protein suspension was sedimented through a 50% sucrose gradient. HIV-1 CA was detected by western blotting of whole lysates, post-sedimentation pellets and supernatants (materials that did not enter the sucrose cushion). The mature CA (24 kDa) band was quantitated for the blot showing the pellet fractions and quantitation data are shown expressed as relative values.

**Figure 6:**
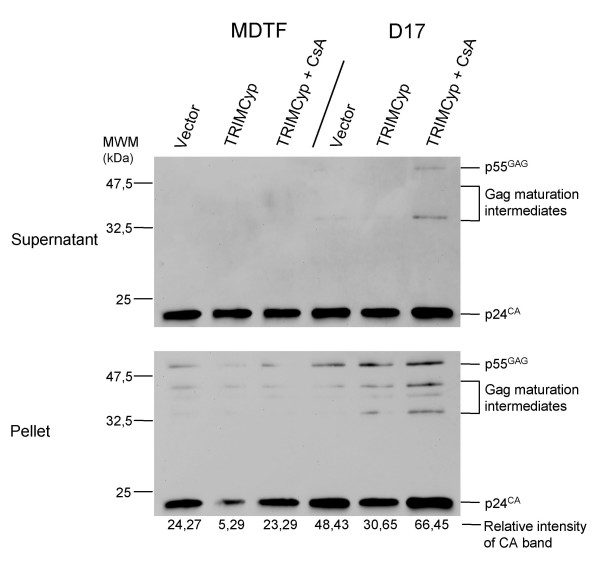
**Fate-of-capsid assay**. MDTF and D17 cells expressing TRIMCyp, and control cells were infected with HIV-1 VLPs as in Fig. 5. The cells expressing TRIMCyp were infected in the presence or not of cyclosporine (5 μM). CA was detected in post-sedimentation pellets and supernatants. Pellet CA was quantitated as in Fig. 5.

Restriction of both HIV-1 and N-MLV by TRIM5α has been associated with a loss of particulate CA [14,20]. Post-entry particulate CA is believed to be a marker of viruses not yet disassembled, as disassembly of the retroviral core leads to increased CA solubility. Using a 50% sucrose cushion, we separated particulate CA from soluble CA following HIV-1 virus-like particles (VLPs) infection of MDTF and D17 cells expressing TRIM5α_rh _or TRIMCyp. Examination of CA in whole lysates and in soluble "supernatant" fractions revealed a larger amount of CA in D17 cells compared with the MDTF cells (Fig. 5). Presumably, this could be due to more efficient virus entry in the D17 cells. Uncleaved Gag proteins and Gag maturation intermediates were detected in whole lysates and in some pellets, but this observation did not fit any obvious trend and had low reproducibility (not shown). As expected, there was a decrease (6-fold) in particulate CA in the MDTF-TRIM5α_rh _cells, compared with the control MDTF cells. The same phenotype was observed in the MDTF-TRIMCyp cells, indicating that TRIM5α and TRIMCyp, despite differences in the CA-binding region, inhibit retroviral replication through similar mechanisms. We also noted that the decrease in particulate CA was not accompanied by an obvious increase in soluble CA (Fig. 5 and Fig. 6). In the D17 cells, TRIM5α_rh _and TRIMCyp both decreased the levels of particulate HIV-1 CA compared with the control cells, but the magnitude of the effect was significantly lesser than in the MDTF cells.

Both TRIMCyp-mediated restriction of HIV-1 and enhancement of HIV-1 replication by CsA are more efficient in the MDTF cells compared with the D17 cells (Fig. 3 and 4). Thus, we examined the effect of CsA on the levels of particulate CA in MDTF-TRIMCyp and D17-TRIMCyp cells (Fig. 6). Like before, the decrease in particulate CA caused by TRIMCyp was more acute in the MDTF cells compared with the D17 cells (5-fold versus 1.6-fold). In addition, CsA restored wild-type levels of particulate CA in both cell types, although, as expected, the magnitude of this effect was greater in the MDTF cells.

**Figure 7:**
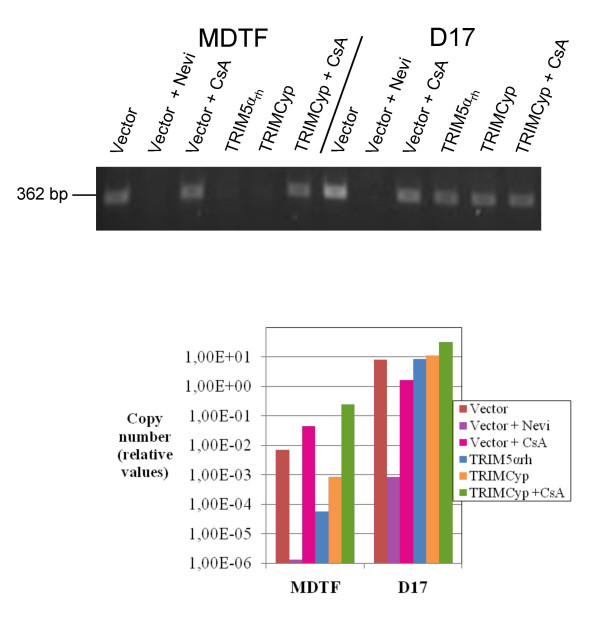
**Retroviral cDNA synthesis**. MDTF or D17 cells expressing the indicated TRIM5 orthologues were infected for 12 hours with HIV-1_GFP _at a MOI yielding about 20% infected cells for the control cells. In addition, infection of cells expressing TRIMCyp was carried out in the presence or absence of 5 μM cyclosporine, and infection of control cells was done in the presence or absence of the reverse transcriptase inhibitor nevirapine (80 μM). Top panel, total cellular DNAs were extracted and an aliquot of each DNA sample was subjected to a 30-cycle PCR amplification using ODNs annealing to GFP sequences. PCR products were separated on an agarose gel and revealed with ethidium bromide. Bottom panel, as above but HIV-1_GFP_-specific DNAs were quantitated by real-time PCR, using dilutions of a plasmid containing the GFP sequence as a standard.

TRIM5α_rh _and TRIMCyp both inhibit HIV-1 cDNA accumulation in their cognate species and this phenotype is maintained upon expression in non-primate cells. We used standard PCR and real-time PCR to analyze the levels of HIV-1 cDNA after a 12-hours HIV-1_GFP _infection of MDTF and D17 cells expressing TRIM5α_rh _or TRIMCyp (Fig. 7). The oligodeoxynucleotide pair used amplified a sequence within the GFP cDNA. Compared with control cells, both TRIM5α_rh _and TRIMCyp caused a sharp decrease in the accumulation of viral cDNA in the MDTF cells. As expected, CsA rescued HIV-1 cDNA synthesis to near-normal levels. On the other hand, TRIM5α_rh _and TRIMCyp caused little or no decrease in HIV-1 cDNA levels in D17 cells and CsA had little or no effect on the levels of cDNA in the D17-TRIMCyp cells (Fig. 7).

**Figure 8:**
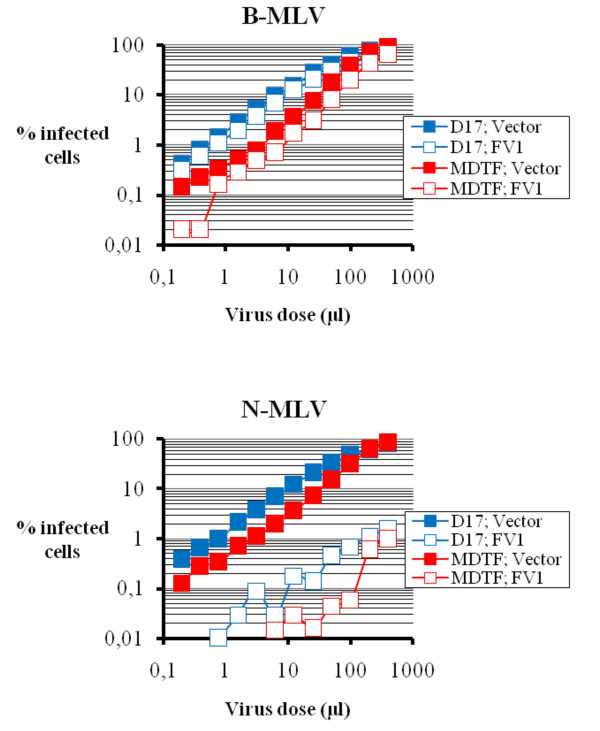
**Restriction by Fv1**. MDTF or D17 cells, expressing Fv1^b ^or not (control cells), were infected with multiple dilutions of N-MLV and B-MLV vectors expressing GFP. The percentage of infected cells was determined by flow cytometry 2 days later.

Fv1, the murine retroviral restriction factor described and cloned decades ago [40,41], also targets incoming retroviruses at an early post-entry step. Although *fv1 *is related to the *gag *region of murine endogenous retroviruses and bears no immediate similarities to *TRIM5*, residues in MLV CA proteins are determinants in both Fv1 and TRIM5α-mediated restrictions [42]. Consequently, Fv1 and TRIM5α compete with one another for the binding to putative restriction targets when co-expressed in the same cells [43]. We transduced the N-MLV-targeting Fv1^b ^in both D17 and MDTF cells and monitored the effect of its expression on the replication of N-MLV and, as a control, B-MLV (Fig. 8). As expected, Fv1^b ^strongly inhibited N-MLV in the MDTF cells (more than 100-fold) while it had little effect on B-MLV. Restriction of N-MLV in D17-Fv1^b ^cells was efficient, albeit slightly less so than in MDTF-Fv1^b ^cells. Thus, loss of restriction activity in D17 cells seems to be specific to TRIM5α.

## Discussion

The mechanism by which TRIM5α and TRIMCyp intercept and inhibit incoming retroviruses is incompletely understood. TRIM5α is able to trimerize in cells, and it is probably in this form (or as a multimer of higher complexity) that it recognizes its viral target [15,17]. This initial interaction is followed by the disappearance of particulate CA but not soluble CA. The loss of particulate CA has been attributed to an acceleration of viral uncoating in restrictive conditions [14,20]. However, as observed here and by others [14], the decrease in particulate HIV-1 CA in restrictive conditions is not necessarily accompanied by an increase in soluble CA. Thus, it remains possible that incoming retroviral cores are not disassembled faster under TRIM5α/TRIMCyp restriction but instead are specifically targeted to a degradation pathway. Accordingly, pharmacological approaches have revealed a role for the proteasome in the restriction mediated by TRIM5α [21,22]. Of course, the two models are not mutually exclusive, as proteasome-mediated degradation might well follow premature decapsidation.

We find retroviral restrictions mediated by either TRIM5α or TRIMCyp (but not Fv1) to be poorly efficient in the canine cells D17. These results confirm and extend previous findings by Saez and colleagues [38]. The restriction defect did not appear to be caused by poor expression or mislocalization of TRIM5α or TRIMCyp. Consistent with the HIV-1_GFP _transduction data, TRIM5α and TRIMCyp had little effect on the accumulation of HIV-1 cDNA in D17 cells. In addition, TRIM5α and TRIMCyp induced the disappearance of HIV-1 particulate CA at relatively low rates in D17 cells, compared with the MDTF cells. Therefore, D17 cells provided a poor environment for the restriction. We hypothesize that a cellular factor important for the activity of TRIM5α and TRIMCyp is not functional or is expressed at low levels in these cells. The missing factor might be important for TRIM5 multimerization or for its interaction with the proteasome. Conversely, a dominant negative factor might be expressed in the D17 cells. That both N-MLV and HIV-1 were less restricted in D17 cells implies that CypA is not relevant to the observed phenotype. Reciprocally, it is unlikely that the SPRY/B30.2 domain of TRIM5α is relevant to its loss of function in the D17 cells, since a similar effect was observed with TRIMCyp.

## Conclusion

The canine D17 cells offer a cellular context that is unfavorable to the restriction mechanism mediated by TRIM5α and TRIMCyp. This cell line may thus represent a unique opportunity to isolate and characterize cellular genes regulating retroviral restrictions.

## Methods

### Plasmid DNAs

pMIP-TRIM5α_rh_-FLAG, pMIP-TRIM5α_AGM_-FLAG, pMIP-TRIM5α_hu_-FLAG, and pMIP-TRIMCyp-FLAG express C-terminal FLAG tagged versions of cDNAs amplified respectively from rhesus macaque FRhK4 cells, African green monkey Vero cells, human TE671 cells, or owl monkey OMK cells, and were generous gifts from Jeremy Luban [39]. pCLNCX-Fv1^b ^[43], which encodes both Fv1^b ^and the red fluorescent protein (RFP), was a kind gift of Greg Towers (University College, London). pMD-G, pΔR8.9, pTRIP-CMV-GFP, pCL-Eco, pCIG3N, pCIG3B and pCNCG have all been extensively described before [44-49].

### Cells and virus production

Human embryonic kidney 293T, human cervical epithelial carcinoma cells HeLa, *mus dunni *tail fibroblasts (MDTF; a gift from Jeremy Luban) and canine osteosarcoma D17 cells (a kind gift from Monica Roth) were all grown in DMEM medium supplemented with 10% fetal bovine serum and antibiotics. All viruses used in this study were produced through transient transfection of 293T cells using polyethylenimine. For that, a mixture of the appropriate DNAs diluted in 1 ml of DMEM without serum or antibiotics was mixed with 45 μl of a 1 mg/ml solution of polyethylenimine (Polysciences). This transfection mix was then added to 70% confluent 293T cells in a 10-cm tissue culture dish. The next day, cells were PBS-washed once and put back in culture in fresh medium. 2 days after transfection, virus-containing supernatants were collected, clarified by low-speed centrifugation and stored in 1-ml aliquots at -80°C.

To produce the CLNCX and MIP vectors used to transduce *fv1*^b ^and the various *TRIM5 *alleles, the transfection mix included 10 μg of pCL-Eco, 5 μg of pMD-G, and 10 μg of the appropriate pMIP or pCLNCX construct. To produce the N-MLV_GFP _and B-MLV_GFP _vectors, the transfection mix included 10 μg of pCIG3 N or B, 5 μg of pMD-G, and 10 μg of pCNCG. To produce the HIV-1_GFP _vector, cells were transfected with 10 μg of pΔR8.9, 5 μg of pMD-G, and 10 μg of pTRIP-CMV-GFP.

### TRIM5-expressing cell lines

HeLa and D17 cells were plated at 300,000 cells per well and MDTF cells were plated at 140,000 cells per well in 6-well plates. The next day, supernatants were aspirated and replaced with MIP-TRIM5α or MIP-TRIMCyp vector preparations (2 ml per well). 2 days later, cells were placed in medium containing 1 μg/ml (HeLa, D17) or 3 μg/ml (MDTF) of puromycin (EMD Biosciences). These puromycin concentrations were determined to kill all sensitive cells after one or two days of treatment. Puromycin selection was allowed to proceed for 4 days, and then again periodically during the course of this work. Expression of the transduced TRIM5 cDNAs was analyzed by western blotting, using antibodies directed against the FLAG epitope (mouse monoclonal; Sigma) or actin (goat polyclonal; Santa Cruz).

### Viral challenges

Cells were plated at 25,000 cells (HeLa, D17) or 10,000 cells (MDTF) in 0.4 ml per well of 24-well plates. Cells were infected the next day with HIV-1_GFP_, N-MLV_GFP_, or B-MLV_GFP _vectors. When CsA (Sigma) or nevirapine were used, they were added 15 min prior to the virus. Cell supernatants were replaced with fresh medium without drugs 16 h after infection. 2 days after infection, cells were trypsinized and fixed in 2% formaldehyde-PBS. Flow cytometry was done on a FC500 MPL instrument (Beckman Coulter) using the CXP software for analysis. Intact cells were identified based on light scatter profiles, and only those cells were included in the analysis. Ten thousand cells per sample were processed, and cells positive for GFP expression were gated and counted as a percentage of total intact cells. Cells expressing Fv1^b ^and RFP were first gated for RFP expression and infected cells were computed as % of cells expressing both RFP and GFP among all RFP-positive cells. False-positive results were insignificant, as shown by controls corresponding to uninfected cells (not shown).

### IF microscopy

Cells were plated at 24,000 (MDTF) or 50,000 (D17) on LabTek II four-chamber slides (LabTek). The next day, cells were washed with PBS, fixed for 30 min in 4% formaldehyde-PBS, washed three times in PBS and permeabilized with 0.1% Triton X-100 for 2 min on ice. Cells were then washed again with PBS and treated with 50 mM NH_4_Cl (in PBS) for 10 min at RT. Then, cells were washed 3 times in PBS and treated with 10% normal goat serum (Vector laboratories) for 30 min at RT. This saturation step was followed by incubation with an antibody against FLAG (M2 mouse monoclonal; Sigma) at a 1:400 dilution in PBS with 10% normal goat serum. Fluorescent staining was done using an Alexa488-conjugated goat anti-mouse antibody (Molecular Probes) at a 1:500 dilution. Cells were washed 4 times in PBS before mounting in Vectashield (Vector Laboratories). Hoechst33342 (0.8 μg/ml; Molecular Probes) was added along with the penultimate PBS wash to reveal DNA. Pictures were generated using a Olympus BX-60 microscope with the Image-Pro Express software.

### Fate-of-capsid assay

The protocol used was adapted from Stremlau et al [14]. Cells were plated at 80% confluence in 10-cm culture dishes. 12 hours later, they were layered with 8 ml of HIV-1 VLPs, which is a high MOI (equivalent to 50–80% infected control cells by HIV-1_GFP_). VLP infections were performed in the presence or absence of nevirapine (80 μM) or CsA (5 μM). 4 hours later, supernatants were replaced with fresh media containing the appropriate drugs and the cells were put back in culture for an additional 2 hours. Cells were then lysed in 1.5 ml of a hypotonic lysis buffer (100 μM Tris-Cl pH8.0, 0.4 mM KCl, 2 μM EDTA) containing a protease inhibitor mix (Sigma). After Dounce homogenization (15 strokes) and clarification by low-speed centrifugation, 50 μl of the lysate were saved ("whole lysate"), and 1 ml was layered on top of a 50% sucrose cushion prepared in PBS. Particulate CA was sedimented by ultracentrifugation using a Beckman SW41Ti rotor. The centrifugation was carried in Beckman Ultraclear tubes for 2 hours at 32,000 rpm and at 4°C. Following this step, 200 μl of the supernatants were carefully transferred to a fresh tube and lysed in SDS sample buffer. Remaining supernatant and sucrose cushions were discarded by carefully inverting the tubes, and pellets were resuspended in 50 μl of SDS sample buffer. Equal volumes of whole cell lysate, supernatant, and pellet fractions were processed for western blotting using a anti-CA mouse monoclonal antibody (clone 183; a gift of Jeremy Luban)

### Monitoring HIV-1 cDNA synthesis

50,000 cells (D17) or 20,000 cells (MDTF) were plated in 0.4 ml per well in 24-well plates. 12 hours later, cells were infected with 10 μl HIV-1_GFP _that had been treated with DNase I (NEB; 23 U/ml of virus preparation) for 10 min at 25°C. Cells were washed with PBS and trypsinized after 12 hours of infection. Total cellular DNA was extracted using the DNeasy kit (Qiagen) and digested for one hour at 37°C with Dpn1 to further reduce contamination of the samples with plasmid DNA. Aliquots (5 μl out of 200 μl) of each sample were submitted to a 30-cycle PCR analysis using the following oligodeoxynucleotides: GFPs, 5'-GACGACGGCAACTACAAGAC and GFPas, 5'-TCGTCCATGCCGAGAGTGAT. PCR products were separated on a 2% agarose-TAE gel, and revealed with ethidium bromide staining. For real-time PCR analysis, 2 μl of each DNA preparation were subjected to a 45-cycle PCR in 20 μl total volume containing 10 μl of QuantiTect SYBR Green PCR master mix (Qiagen). Amplification curves were analyzed with Light Cycler relative quantification software v1.0, and quantifications were determined relative to dilutions of pTRIP-CMV-GFP.

## Competing interests

The author(s) declare that they have no competing interests.

## Authors' contributions

LB and JB designed the study. JB and AB performed experiments. LB and JB drafted the manuscript. All authors read and approved the final manuscript.
